# Spinal schwannoma missed on lumbar MRI: a case report

**DOI:** 10.3389/fmed.2026.1741404

**Published:** 2026-02-26

**Authors:** Pengfei Zhang, Ziyuan Zhang, Wenqin Sun

**Affiliations:** 1 Department of Anesthesiology, Changde Hospital, Xiangya School of Medicine, Central South University (The First People’s Hospital of Changde City), Changde, Hunan, China; 2 Central South University, Changsha, Hunan, China

**Keywords:** case report, lumbar disk herniation, magnetic resonance imaging, nerve root sheath cyst, selective nerve root block, spinal schwannoma

## Abstract

A 55-year-old male presented with 9 months of left buttock and lower limb pain, initially misdiagnosed as lumbar disk herniation. Unsuccessful lateral recess block and physical therapy prompted further evaluation. Lumbar magnetic resonance imaging (MRI) revealed a right L5 nerve root cyst, yet symptoms worsened. Examination showed left paraspinal tenderness, grade IV left lower limb weakness, hyperesthesia, and positive straight leg raise tests. Selective nerve root block localized the lesion to the left L5 root; contrast-enhanced MRI identified an L4-5 facet joint cyst with inflammation. Arthroscopic-assisted Uni-portal Spinal Surgery (AUSS) endoscopic resection under general anesthesia was performed, with pathology confirming schwannoma. Postoperatively, pain intensity (numerical rating scale, NRS) decreased from 7 to 2 within 24 h; discharge occurred on day 6. At 2-month follow-up, symptoms resolved completely. This case underscores the diagnostic pitfalls of lumbar schwannoma (mimicking disk herniation/cysts) and highlights the roles of nerve root blocks, contrast MRI, and histopathology. AUSS endoscopy achieved definitive management.

## Introduction

Spinal nerve sheath tumors, or schwannomas, have an estimated annual incidence of 3 to 7 cases per million individuals, with a predilection for adults aged 40 to 60 years (1). These tumors arise from Schwann cells, the primary constituents of the nerve sheath, and are predominantly located in the intradural extramedullary compartment, though intramedullary or extradural occurrences are also documented. Typically, these lesions manifest as well-encapsulated, oval-shaped masses adherent to one or two nerve roots. Clinically, spinal nerve sheath tumors exhibit a slow progression, often leading to gradual deterioration of neurological function. Schwannoma tumorigenesis is driven by diverse molecular alterations, including canonical mutations in NF2, SMARCB1, and LZTR1 on chromosome 22q, as well as more recently identified alterations involving ARID1A, ARID1B, DNA damage response (DDR) genes, and the fusion oncogenes SH3PXD2A: HTRA1 and NONO: TFE3 (2). Despite being benign, spinal schwannomas may cause spinal cord compression and subsequently acute or chronic neurological dysfunction (3). MRI is the gold standard imaging modality for detecting spinal schwannomas, offering superior soft tissue contrast to delineate tumor extent, nerve root involvement, and associated cord compression (4). In this report, we present a case of a lumbar nerve sheath tumor characterized by lumbosacral and leg pain as the primary symptoms. Our objective is to elucidate the clinical presentation of this condition and mitigate the risk of misdiagnosis or delayed recognition.

## Case report

**Figure 1:**
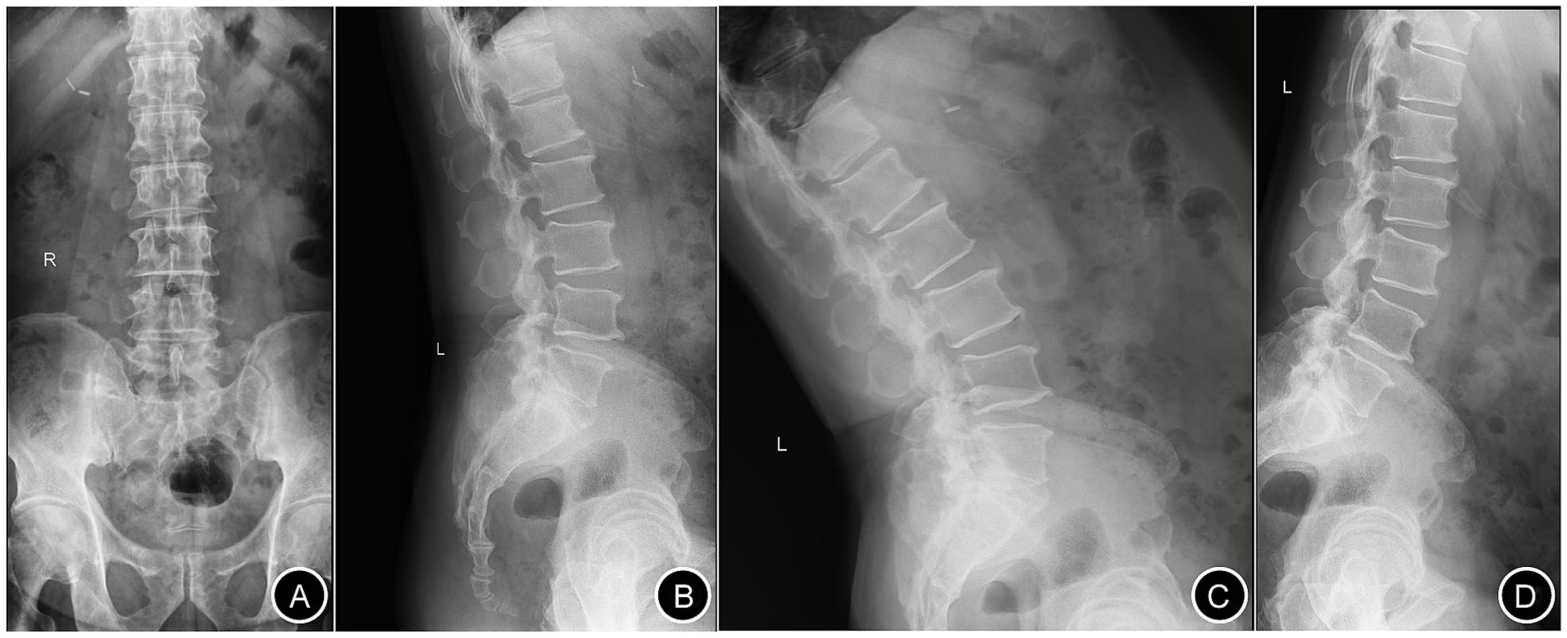
Lumbar spine X-ray. Panels **(A–D)** represent the anteroposterior, lateral, hyperextension, and hyperflexion views, respectively.

**Figure 2:**
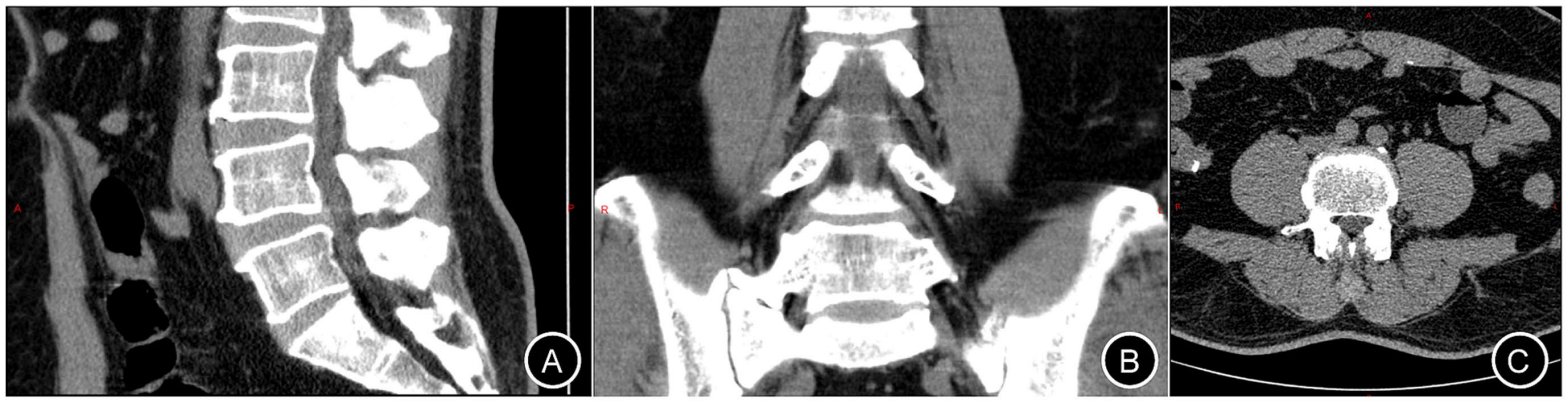
Lumbar intervertebral disk CT. Panels **(A–C)** correspond to the sagittal, coronal, and axial planes, respectively.

**Figure 3:**
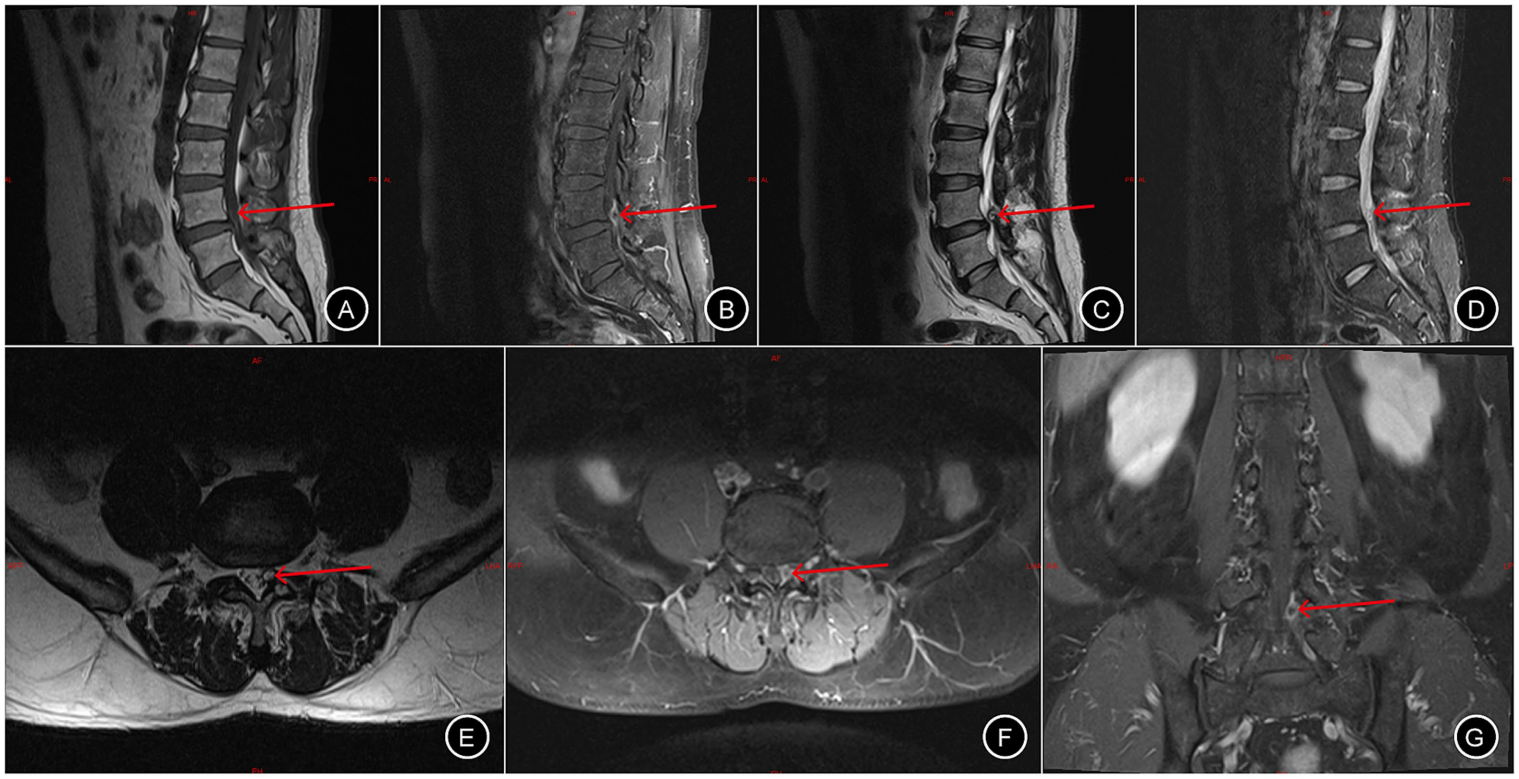
Lumbar intervertebral disk MRI with and without contrast. Panels **(A–G)** depict the sagittal T1-weighted, sagittal T1-weighted post-contrast, sagittal T2-weighted, sagittal T2-weighted fat-suppressed, axial T1-weighted, axial T1-weighted post-contrast, and coronal T1-weighted post-contrast, respectively. The red arrow indicates the schwannoma.

**Figure 4:**
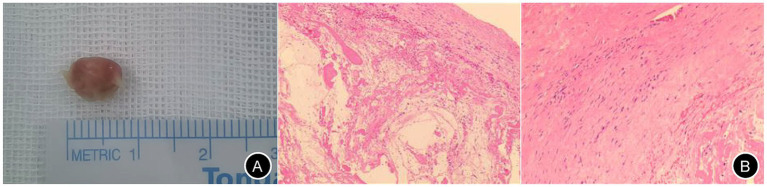
Gross and microscopic specimens of the schwannoma. Panel **A** displays the gross appearance of the tumor, while Panel **B** shows the microscopic features (left: 10 × 4 magnification; right: 10 × 10 magnification).

**Figure 5:**
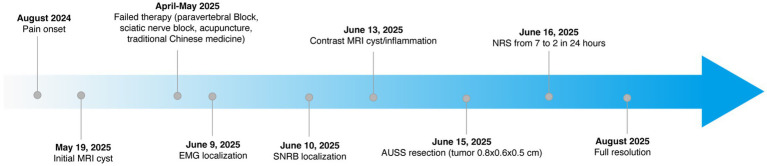
Timeline of clinical events.

A 55-year-old male (height: 174 cm; weight: 88 kg) was admitted to Changde Hospital, Xiangya School of Medicine, Central South University(The first people’s hospital of Changde city) for left buttock and lower limb pain lasting 9 months. The pain, described as a persistent, moderate, dull ache localized to the left buttock, posterior thigh, and posterolateral calf, worsened with walking and lacked a relieving posture. The pain did not occur at night. The patient had no familial history of genetic diseases and no remarkable past medical history. Physical examination revealed tenderness and radiating pain at the L4/5 intervertebral space, radiating to the left posterior thigh, posterolateral calf, and dorsum of the left foot. Positive left straight leg raise test at 40° and positive reinforcement test. Lumbar X-rays revealed degenerative lumbar changes and sacral lumbarization, with no evidence of spondylolisthesis or instability (Figure 1). Lumbar disk computed tomography (CT) showed osteophytosis and bulging of the L3-L5 intervertebral disks, without spinal canal stenosis (Figure 2). Electromyography(EMG) of the lower limbs indicated chronic neurogenic damage in the left L5-innervated muscles, suggestive of lumbosacral nerve root involvement. Selective nerve root block (SNRB) of the left L5 nerve root under ultrasound and neurostimulation guidance resulted in significant pain relief, implicating the left L5 nerve root as the source of pathology. Lumbar MRI (plain and contrast-enhanced) demonstrated a cystic lesion at the left L5 nerve root (Figure 3). Based on the patient’s symptoms, physical signs, and imaging findings, a left L5 radiculopathy is suspected. Following comprehensive preoperative evaluation and exclusion of surgical contraindications, the patient underwent AUSS (Arthroscopic-assisted Uni-portal Spinal Surgery) endoscopic intraspinal lesion resection under general anesthesia on June 15, 2025. During the procedure, a well-circumscribed, cylindrical tumor (0.8 cm × 0.6 cm × 0.5 cm) was identified adherent to the dorsal aspect of the dura and the left L5 nerve root, causing significant compression (Figure 4). The tumor was well-demarcated from the nerve roots, with a complete capsule. It was soft in consistency, pale yellow, and semi-translucent in appearance. The tumor was completely excised and sent for pathological examination. On the second day after surgery, the patient’s pain in the left buttock and left lower limb improved significantly, with the Numerical Rating Scale (NRS) score decreasing from 7 to 2 points. Research has shown that, in patients with chronic musculoskeletal pain, a reduction of one point or a reduction of 15.0% in the Numerical Rating Scale (NRS) represents the minimal clinically important difference (MCID)—the smallest change perceived as clinically meaningful—while a NRS change score of −2.0 or a percent change score of −33.0% is generally considered a “much better” improvement (5). This marked reduction in pain indicated excellent surgical outcomes. The postoperative pathological diagnosis (Figure 4B) revealed a ‘spinal canal’ spindle cell tumor, consistent with a schwannoma. A postoperative lumbar CT scan showed no abnormalities. During the 2-month follow-up, the patient’s left buttock and left lower limb pain symptoms had almost completely resolved (Figure 5). Patient reported: “Pain relief allowed return to work.”

## Discussion

Spinal schwannomas originate from Schwann cells harboring NF2 gene mutations. These tumors may arise from any component of spinal nerves, including ventral/dorsal roots, rami, or sympathetic structures. Clinical manifestations include radicular pain, numbness, motor weakness due to nerve root irritation, gait disturbances, and incontinence secondary to spinal cord compression (6). The insidious onset and nonspecific symptomatology often lead to misdiagnosis as lumbar disk herniation (7), neurofibroma (8), or spinal hematoma (9).

The clinical differentiation between lumbar disk herniation(LDH) and spinal canal tumors is critical yet challenging due to overlapping symptoms. LDH typically presents with activity-aggravated pain that alleviates with rest and tends to recur, whereas spinal canal tumors often manifest as progressive pain exacerbated at rest or during nighttime, with temporary relief upon activity.

Advancements in modern medical imaging, particularly CT and MRI, have revolutionized the diagnosis of spinal and intraspinal pathologies. However, imaging alone achieves an accuracy of only approximately 80%, and overreliance on these modalities can lead to misdiagnosis (10). Conventional spinal X-rays and CT scans lack specificity for intraspinal tumors, as they fail to distinguish soft tissues such as muscles, ligaments, and neural structures. In contrast, MRI has emerged as the gold standard for diagnosing intraspinal schwannomas (11). It not only delineates the lesion’s location, extent, and morphological features but also reveals spinal cord and nerve root displacement, compression, and edema, enabling preliminary tumor characterization. On MRI, schwannomas are commonly isointense on T1 weighted images and hyperintense on T2 weighted images with heterogeneous enhancement after intravenous administration of contrast agent. However, due to inflammation and neovascularization, LDH may exhibit a ring enhancement phenomenon (either completely or partially surrounding the herniation) on contrast-enhanced MRI. Recent advancements, such as MRI-based deep learning classification models, offer automated diagnostic capabilities (12). Additionally, preoperative MRI can classify the origin of spinal schwannomas, with contrast-enhanced MRI demonstrating marked enhancement of solid tumor components while leaving cystic or necrotic areas unenhanced (13). Further refinement is achieved through magnetic resonance neurography, which provides superior visualization of extradural neural structures and their anatomical relationships compared to conventional MRI.

To avoid or reduce misdiagnosis of lumbar spinal schwannomas, the focus should be on both clinical manifestations and imaging features. In cases where clinical findings and imaging results are discordant, multidisciplinary consultation is indispensable for achieving a definitive diagnosis.

This case illustrates how diagnostic errors may result from both technical limitations in imaging and cognitive factors. Overreliance on radiology reports, especially when they fail to address technical limitations, can lead to overconfidence and premature diagnostic closure, a form of cognitive bias. The reassessment of imaging in the context of evolving clinical signs serves as a critical safeguard against such errors. Pain physicians, with training in holistic assessment, increasing familiarity with imaging interpretation and high proficiency in nerve block procedures, are uniquely positioned to identify diagnostic mismatches and challenge initial assumptions. Furthermore, this case underscores the importance of correlating neurological findings with imaging results. When discrepancies arise between clinical findings and radiologic interpretations, further investigation should be pursued without delay.

Surgical resection remains the primary treatment for spinal schwannomas. Recently, a novel surgical approach—AUSS (Axial Unilateral Single-portal Split Spine endoscopy)—has emerged, evolving from the unilateral biportal endoscopic (UBE) technique. AUSS integrates the traditional dual-incision, single-sided dual-channel UBE approach into a single portal. By incorporating an endoscope into conventional open surgery, AUSS offers the advantages of an expansive visual field, ample working space, and compatibility with a wide range of spinal surgical instruments and techniques, combining the benefits of minimally invasive access with the familiarity and versatility of open procedures (14). The MRI coronal view confirms that the tumor in this patient is located dorsal to the left L5 nerve root, suggesting the AUSS technique as a feasible option. The advantage of the AUSS technique for this case lies in performing laminectomy, followed by removing the proliferative ligamentum flavum to fully expose the nerve root and dural sac. In addition to the above, emerging evidence suggests that progranulin (PGRN), a multifunctional protein endowed with neuroimmunomodulatory and oncogenic properties, may represent a promising therapeutic target for schwannoma (15).

In summary, spinal schwannomas are diagnostically challenging due to their rarity and nonspecific presentation. Firstly, a thorough clinical assessment, including detailed history-taking and comprehensive physical examination, is critical to identify subtle but indicative features of spinal schwannomas. The hallmark clinical manifestation of schwannoma is progressive lower limb pain exacerbated by physical activity, often unaccompanied by discernible abnormalities on physical examination. Secondly, contrast-enhanced MRI is strongly advocated as the imaging modality of choice to enhance diagnostic accuracy, with hydrography as an alternative for select cases. The spinal schwannomas appears hypointense or isointense on T1-weighted images, hyperintense on T2-weighted images, and demonstrates heterogeneous enhancement after intravenous contrast administration isointense on T1 weighted images and hyperintense on T2 weighted images with heterogeneous enhancement post-contrast. Thirdly, for diagnostically equivocal cases, SNRB and multidisciplinary consultation serve as an indispensable step to resolve uncertainty, optimize management strategies, and improve patient outcomes. Moreover, AUSS offers an effective, minimally invasive approach for the diagnosis and treatment of lumbar schwannoma.

## Data Availability

The original contributions presented in the study are included in the article/Supplementary material, further inquiries can be directed to the corresponding author.
